# Characteristics of the patients with delaminated rotator cuff tear

**DOI:** 10.1051/sicotj/2018022

**Published:** 2018-07-06

**Authors:** Satoshi Iwashita, Hiroshi Hashiguchi, Atsushi Okubo, Minoru Yoneda, Shinro Takai

**Affiliations:** 1 Department of Orthopaedic Surgery, Nippon Medical School Hospital, 1-1-5 Sendagi, Bunkyo-ku, Tokyo 113-8602 Japan; 2 Department of Orthopaedic Surgery, Nippon Medical School Chiba Hokusoh Hospital, 1715 Kamakari, Inzai, Chiba 270-1694 Japan

**Keywords:** Rotator cuff tear, Delamination, Cuff integrity, Arthroscopic rotator cuff repair

## Abstract

*Purpose*: The purpose of this study was to analyze factors relating to delamination in full-thickness rotator cuff tears.

*Methods*: 126 patients with full-thickness rotator cuff tears treated by arthroscopic rotator cuff repair were the subjects of this study. There were 52 females and 74 males whose average age was 64.2 years. Fifty-three patients had history of trauma. The average duration of disorder was 29.5 weeks. Nineteen patients were diagnosed with diabetes. On types of the tears, small tear was observed in 59 patients, medium tear in 47 patients, large tear in 6 patients, and massive tear in 14 patients. The average size of tear was 1.98 cm. Delamination of the torn cuff was observed in 45 patients. Factors compared between the patients without delamination and those with delamination were as follows: gender and age of the patients, history of trauma, duration of disorder, diabetes, smoking, size and number of rotator cuff tears.

*Results*: The delamination rate of the smoking patients was significantly higher than non-smoking patients. The delamination rate of patients with more than two tendon tears was significantly higher than those with only one tendon tear. The average size of tear with delamination was significantly larger than that of tear without delamination. The other factors were not related to delamination.

*Conclusions*: This study suggests that smoking, size of tear and number of torn cuffs are associated with delamination. The progression of torn cuff, anatomical features and nicotine of smoking affect the causes of delaminated tear of rotator cuff.

## Introduction

Delamination is defined as a separation of the stump of the full-thickness rotator cuff tear into the superficial and deeper layers. Good cuff integrity is a key determinant for successful rotator cuff repair [1]. In addition, cuff integrity may be used, as a predictor of poor treatment outcome when delamination is undiagnosed or when the anatomical repair of the superficial and deep layers is difficult. It is therefore important to understand the pathological features of and surgical techniques for delamination, but no unified view is currently available on these. In this study, we investigated arthroscopic findings of delaminated rotator cuff tears to reveal the pathological features and factors involved in their occurrence.

## Material and methods

Subjects were 126 patients and 126 shoulders (52 women, 74 men) median age 64.2 years (40–80 years) who underwent arthroscopic rotator cuff repair. Operations were performed between April 2011 and March 2014 by a single senior surgeon. This study was a retrospective study. And the surgeon was blinded to some of the patient characteristics the authors were looking at. Of the 126 patients, 53 had a history of injury. Mean disease duration was 29.5 weeks (1–50 months). Medical history revealed the presence of diabetes in 19 patients and smoking history in 18. According to Cofield classification, rotator cuff tears were small in 59 patients, medium in 47 patients, large in 6 patients, and massive in 14 patients, with the mean tear length of 1.98 cm. Clear arthroscopic findings of delamination between the superficial and deep layers were observed in 45 patients. Items evaluated in the study were patient factors such as age, sex, presence of diabetes, history of smoking or injury, duration of disorder, tear type and size, and the number of tendons involved. These items were compared between patients with delamination (delamination group) and those without (non-delamination group). Statistical analysis was performed using the Mann–Whitney *U* test, Chi-square test and Independence test, with the significance set at *p* < 0.05.

## Results

**Figure 1:**
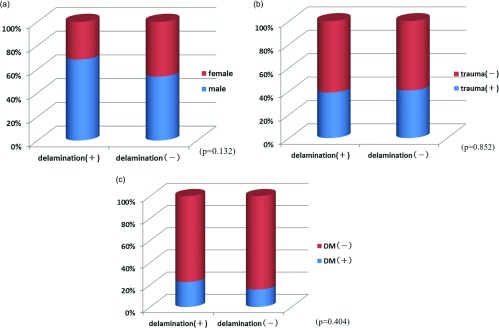
(a) Relation between delamination and gender. (b) Relation between delamination and history of trauma. (c) Relation between delamination and diabetes.

**Figure 2:**
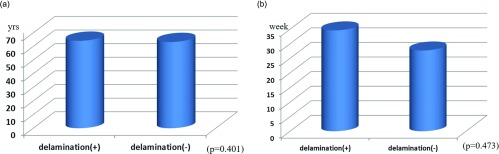
(a) Relation between delamination and age. (b) Relation between delamination and duration of disorder.

**Figure 3:**
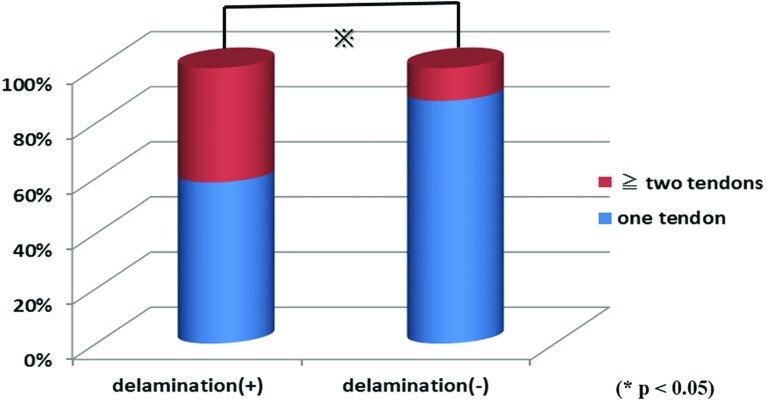
Rate of number of torn tendon.

**Figure 4:**
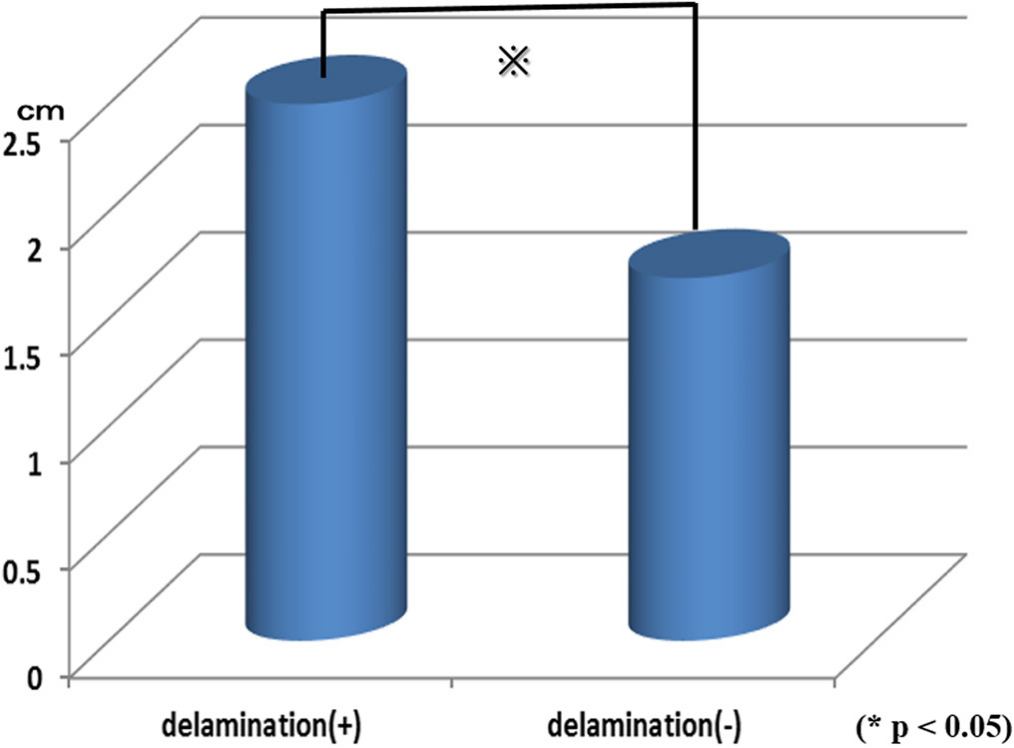
Relation of delamination and width of torn cuff.

**Figure 5:**
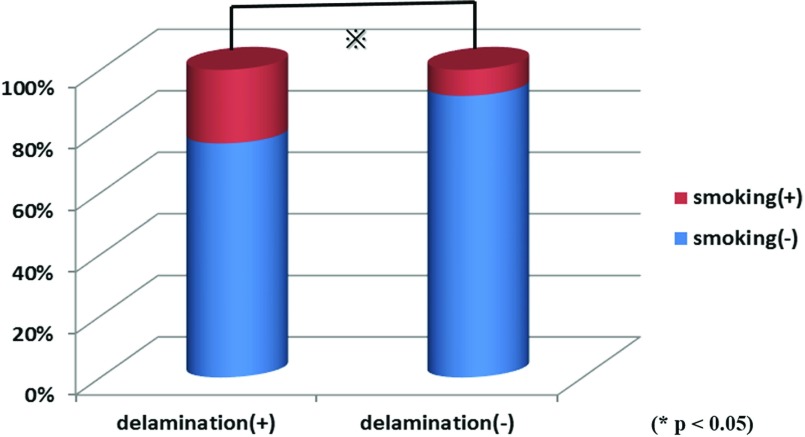
Relation of delamination and smoking.

No significant differences in sex (*p* = 0.132), injury (*p* = 0.852), or diabetes (*p* = 0.404) were observed between the delamination and non-delamination groups (Figures 1a–1c). Mean age was 65.3 years (42–80 years) in the delamination group and 63.9 years (38–78 years) in the non-delamination group, with no significant difference in age between them (*p* = 0.401). Mean condition duration was 33.4 weeks (1–50 months) in the delamination group and 26.7 weeks (1–40 months) in the non-delamination group; again, no significant difference in duration was found between the groups (*p* = 0.473) (Figures 2a and 2b). The proportion of patients with tears in ≥2 tendons was 41.5% in the delamination group, which was significantly higher than the 11.8% in the non-delamination group (Figure 3). There was significant difference in proportion of tear type between two groups and tear size was 2.50 cm in the delamination group, which was significantly higher compared with the 1.69 cm in the non-delamination group (Figure 4). The rate of smoking was 24.4% in the delamination group, which was significantly higher compared with 8.6% in the non-delamination group (Figure 5).

## Discussion

Among full thickness rotator cuff tears, the stump of torn cuff which separates to superficial and deep layer is defined as delamination. Boileau and Flurin indicated that the delamination was negatively associated with tendon healing [2,3]. Although we encounter delamination tears from time to time in arthroscopic rotator cuff repair, the characteristics, pathology, and surgical technique of delamination remain unclear [4]. The frequency of occurrence was 35% in this study, which is comparable with the 38–71% reported in previous studies [3,5,6]. Variations in the frequencies in previous studies were likely due to the use of various surgical procedures, such as arthroscopic surgery and open surgery, and various definitions for delamination, such as the inclusion or exclusion of partial-thickness tears. The frequency of occurrence was low in this study presumably because we defined delamination as full-thickness tears with a clear separation between the superficial and deep layers on arthroscopy.

Matsumoto et al. reported that the clinical characteristic of delamination of the rotator cuff is its high percentage in elderly individuals and in women [6], while Grame et al. observed no age- or sex-related difference in its occurrence [7], demonstrating no unified view on the roles of age or sex in delamination. In this study, the percentage of delamination tears showed no significant association with age, sex, condition duration, injury, or diabetes. However, the percentage was significantly higher in patients with tear in ≥2 tendons of the rotator cuff and in patients with large size tears, the latter of which suggests that delamination is a progressive lesion. First, a tear develops at what Codman describes as the rim rent due to less blood flow and stress concentrated on the joint capsule side at the time of shoulder elevation [8,9]. Thereafter, the tear extends into the rotator cuff remaining on the bursa side due to an increase in subacromial impingement induced by an enlargement in the tear size and due to an increase in stress concentration on the remaining rotator cuff on the bursa side. It is possible that delamination is caused by tears in the superficial and deep layers occurring at different time points. This explains the notable retraction that is normally observed on the joint capsule side of delamination tears compared with the bursa side [7]. The high percentage of delamination in patients with tears in ≥2 tendons of the rotator cuff muscle group suggests the involvement of tissues surrounding the muscle group in the development of delamination. The dorsal muscle fibers of the infraspinatus muscle continue into the second, or superficial, layer of the supraspinatus muscle, while the ventral fibers of the infraspinatus muscle continue into the third, or deep, layer of the supraspinatus muscle. In particular, the supraspinatus and infraspinatus tendons overlap in some areas of the tissue surrounding the rotator cuff [10,11]. Because of the anatomical features of the surrounding tissue, forces generated in the superficial and deep layers may differ by size or direction, resulting in the delamination of the layers by shear force. In this study, the percentage of delamination of the rotator cuff was high among smokers. It has been shown that nicotine intake from smoking and the inactivation of matrix metalloproteinases suppress the formation of bone and inhibit the restoration of soft tissues including the rotator cuff [12–14]. It is therefore possible that smoking contributed to the inhibition of spontaneous repair of the delaminated tears between the superficial and deep layers. The findings in this study also suggest that careful observation is necessary to discover delamination in patients with ≥2 tendon tears, those with large size tears, and in those who smoke. In patients with delamination, it may be necessary to scrape between the layers to enhance the cuff integrity and promote repair and fusion of layers [2,5,10]. The limitation was that this study was a retrospective study, therefore we cannot really know whether these factors indeed contribute or are just associations seen with delamination. In future, we should perform prospective well-powered studies to clarify the nature of this association.

## Summary

-  Factors involved in delamination were investigated.
-  Delamination was observed in 45 (35.7%) of the 126 patients with rotator cuff tears. The percentage of delamination was high in patients with large size tears, those with ≥2 tendon tears, and in those who smoke.
-  In patients with the conditions described above, physicians should examine for delamination carefully and when present, perform a repair that takes into consideration all the layers or consider scraping between the layers to facilitate the fusion of the layers.

## Conflict of interest

The authors declare that they have no conflicts of interest in relation to this article.
